# Molecular mechanisms underlying the high mortality of hypervirulent *Klebsiella pneumoniae* and its effective therapy development

**DOI:** 10.1038/s41392-023-01490-9

**Published:** 2023-05-30

**Authors:** Qi Xu, Miaomiao Xie, Xiaoxuan Liu, Heng Heng, Han Wang, Chen Yang, Edward Wai-Chi Chan, Rong Zhang, Guan Yang, Sheng Chen

**Affiliations:** 1 Department of Infectious Diseases and Public Health, Jockey Club College of Veterinary Medicine and Life Sciences, City University of Hong Kong, Kowloon, Hong Kong China; 2 State Key Lab of Chemical Biology and Drug Discovery and the Department of Food Science and Nutrition, The Hong Kong Polytechnic University, Hom Hung, Kowloon, Hong Kong China; 3 Department of Clinical Laboratory, Second Affiliated Hospital of Zhejiang University, School of Medicine, Zhejiang, Hangzhou China

**Keywords:** Inflammation, Infectious diseases

**Dear Editor**,

*Klebsiella pneumoniae* (*Kp*) has become the most important bacterial pathogen causing high mortality rates in clinical patients due to the continuous evolution to several important variants such as carbapenem-resistant (CR-*Kp*), hypervirulent (hv*Kp*) and both CR and hv *K. pneumoniae* (CR-hv*Kp*). The high mortality caused by clinical hv*Kp* is attributed to the non-response to antibiotic treatment. Therefore, a comprehensive understanding of hv*Kp*-host interaction, especially the hv*Kp*-mediated immune responses, is necessary. Although *Kp*-host interaction has been studied for more than 20 years, most research showed that pro-inflammatory signaling was crucial to *Kp* clearance in the host,^1^ without providing evidence to explain why hv*Kp* causes a high rate of death.

To investigate the underlying mechanisms of hv*Kp* pathogenesis, we first infected mice with 17ZR101, a K2 hv*Kp* clinical strain, and HKU1, a classical *Kp* (c*Kp*) strain, and evaluated the distinct immunological responses elicited in the animals. Antimicrobial susceptibility tests performed on these two strains showed that they were resistant to cefotaxime and meropenem but remained susceptible to amikacin, polymyxin B, and tigecycline (Supplementary Table 1). We found that mice infected by 17ZR101, but not HKU1 or the hv*Kp* control strains HvKP4 and HvKP1088, died within 24 h post-infection (hpi)^2–4^ (Fig. 1a) with a series of tissue damages in various organs (Fig. 1b**;** Supplementary Fig. 1a, b). 17ZR101-infection resulted in significantly higher bacterial burdens in various organs at 12 hpi when compared with HKU1-infected animals (Fig. 1c). Intracellular bacteria counting showed that although 17ZR101 could be readily engulfed, this strain could survive within phagocytes (Fig. 1d). Moreover, we found that the animals infected by a K2-hv*Kp* virulence plasmid-cured strain (17ZR101-PC) all survived (Fig. 1a**;** Supplementary Fig. 1c) with similar tissue damage to those infected by HKU1 (Fig. 1a, b**;** Supplementary Fig. 1a, b), which was reversed by virulence plasmid complementary strain 17ZR101-PC-TC (Fig. 1a), confirming that the virulence plasmid played a critical role in the pathogenesis of hv*Kp* by enabling better survival within the host.

**Fig. 1:**
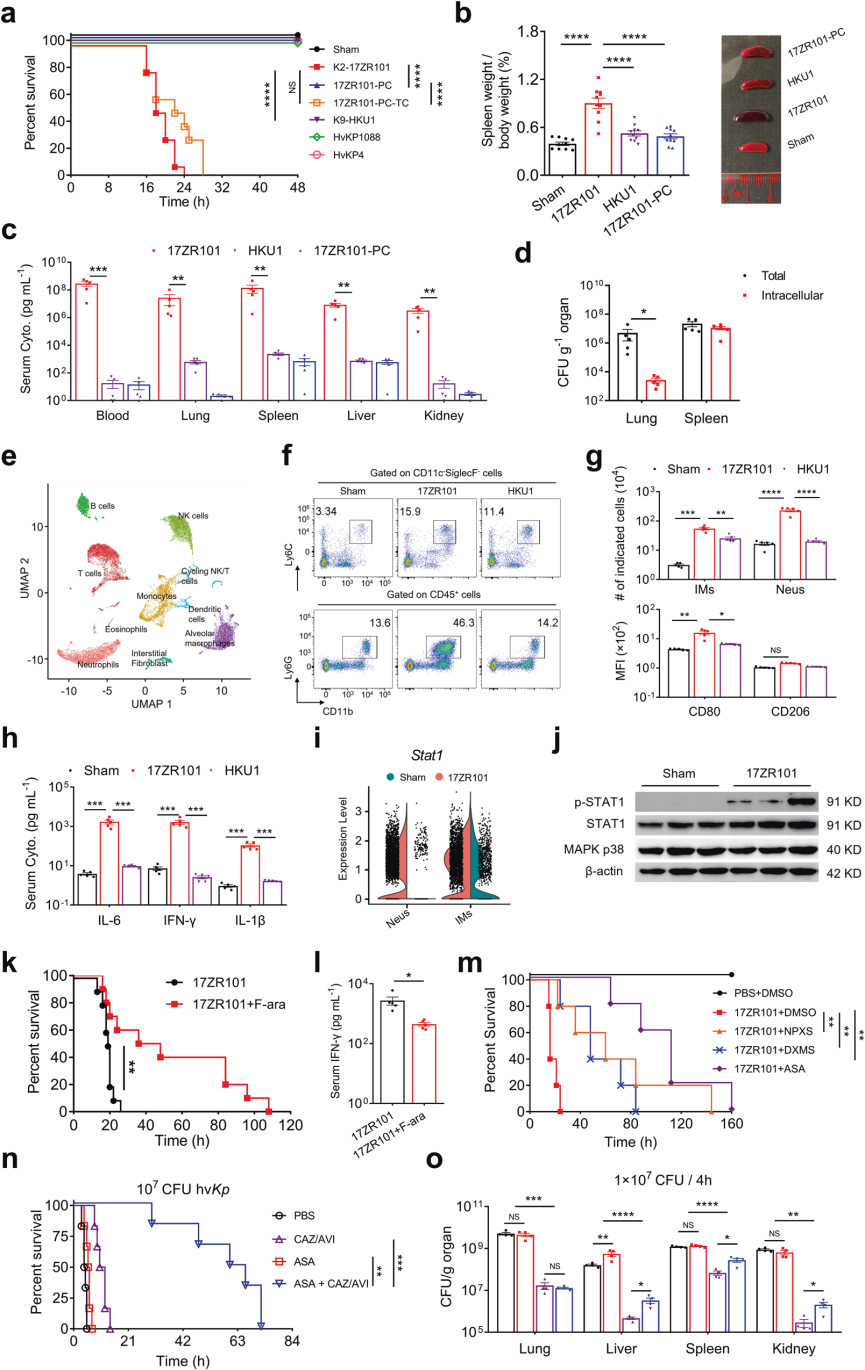
**a** C57BL6 mice were inoculated with 10^4^ CFU of indicated *Kp* strains and the Kaplan–Meier survival curve was recorded. *n* = 10/group. **b** Spleen weight and representative image of spleens harvested from *Kp*-infected mice. **c** Bacterial load in various organs of mice at 12 hpi. *n* = 5/group. **d** Cell suspensions recovered from the lungs and spleens of the test mice at 12 hpi were incubated with 300 μg/mL amikacin for 1 h and the number of CFU was counted. *n* = 5/group. **e** Major clusters and respective cell-type assignments in UMAP. **f** C57BL6 mice were inoculated with 10^4^ CFU of indicated *Kp* strains and IMs and Neus cells recovered from *Kp*-infected lungs were analyzed by flow cytometry. *n* = 5/group. **g** Quantification of IMs and Neus from (**f**) and Mean fluorescence intensity (MFI) of CD80 and CD206 on the surface of IMs. **h** Quantification of serum IL-6, IFN-γ, and IL-1β levels by ELISA. **i** Expression of *Stat1* in Neus and IMs was identified by scRNA-Seq. **j** C57BL6 mice were inoculated with 10^4^ CFU of indicated *Kp* strains and lung sample lysates were prepared for immunoblotting at 12 hpi. Beta-actin was used as a loading control. **k** C57BL6 mice were inoculated with 10^4^ CFU of 17ZR101 hv*Kp* strains and F-ara was administrated at 3 hpi. Survival rate of infected mice was recorded. *n* = 10/group. **l** Analysis of serum IFN-γ levels of mice from (**k**). *n* = 5/group. **m** C57BL6 mice were inoculated with 10^4^ CFU of indicated *Kp* strains and various therapeutic drugs were administrated at 3 hpi. The Kaplan–Meier survival curve of mice was recorded. *n* = 5/group. **n** The survival curve of mice infected with 10^7^ CFU hv*Kp* and these mice were given PBS, CAZ-AVI, ASA, and CAZ/AVI + ASA at 1 hpi. *n* = 6/group. **o** Bacteria burden of various organs from mice infected by 10^7^ CFU hv*Kp* and treated with drugs at 4 hpi. Data were represented as mean ± SEM. NS, not significant; *p < 0.05, **p < 0.01, ***p < 0.001, ****p < 0.0001

Since strain 17ZR101 could be engulfed by phagocytes but was still able to cause severe infections, we hypothesized that the symptoms of infections were caused by the strong and over-active immune responses elicited by hv*Kp*. To characterize the immune landscape in hv*Kp*-infected lungs, we performed the single-cell RNA-Seq (scRNA-seq) to investigate the immune heterogeneity of the infected lung. Using scRNA-seq and an integrated quality control pipeline, we generated a lung atlas that profiled 21,298 cells, including 10,489 from 17ZR101-infected lungs and 10,809 from sham-treated lungs. We visualized the data with dimensionality reduction using uniform manifold approximation and projection (UMAP) and identified 10 major cell types (Fig. 1e**;** Supplementary Fig. 2a). Notably, 17ZR101-infection reduced the frequency of all lymphocyte compartments and alveolar macrophages (AMs) and increased the levels of interstitial macrophages (IMs) and neutrophils (Neus) (Supplementary Fig. 2b, c), indicating that IMs and Neus are the major sources of dysregulated inflammation in 17ZR101 infection. Also, IMs and Neus activation was associated with significant changes in several pathways, related to leukocyte migration, cytokine production, and inflammatory responses (Supplementary Fig. 3a). Reclustering of IMs and Neus revealed an increased M1-like subpopulation (*Aif1*^+^*Ctsb*^+^) (Supplementary Fig. 3b) and a pro-inflammatory neutrophil subset (*Ngp*^+^*Cd177*^+^) (Supplementary Fig. 3c) in 17ZR101-infected lungs. Altogether, these results uncovered that the dysregulation of cytokine production and inflammatory response are the main features of IMs and Neus following 17ZR101 infection.

To validate these scRNA-seq observations, we analyzed the changes in the amount and types of immune cells and signaling molecules of the immune system in the lungs of mice infected by 17ZR101 and observed markedly increased infiltration of IMs (Ly6C^+^CD11b^+^CD11c^−^ SiglecF^−^) and Neus (CD11b^+^Ly6G^+^) in these organs (Fig. 1f, g). Consistent with their microbicidal activity role, studies have identified *Kp* infection triggers the recruitment of IMs.^1,5,6^ The infiltrated IMs were also found to express higher levels of CD80 proteins at their surface, indicating that the proinflammatory M1 polarization process has been enhanced (Fig. 1g). High-throughput RNA sequencing (RNA-seq) analysis of total RNA isolated from the lungs of 17ZR101*-*infected mice further revealed cytokine production, cell chemotaxis, and extracellular matrix organization were depicted during infection by 17ZR101 (Supplementary Fig. 4), confirming that hv*Kp* infection triggered strong inflammatory responses in the host. In addition, 17ZR101-infected mice exhibited over a 100-fold increase in the levels of serum interleukin (IL)-6, interferon (IFN)-γ, and IL-1β (Fig. 1h). Taken together, these findings confirmed the flow cytometry data that 17ZR101 induced M1 polarization and neutrophil chemotaxis, which is characteristic of a cytokine storm.

Among these known macrophage polarization regulation factors, *Stat1* expression (Log2 fold change: 2.700) was found to increase dramatically upon 17ZR101 infection (Supplementary Fig. 5a, b), indicating that this pathway became active during infection. Particularly, our qPCR result confirmed a 5.2-fold increase in the expression of *Stat1* (Supplementary Fig. 5c). More convincingly, *Stat1* was highly expressed in 17ZR101-infected IMs and Neus revealed by scRNA-Seq (Fig. 1i), and up-regulated STAT1 and phosphorylated STAT1 (p-STAT1) by western blotting (Fig. 1j). Flow cytometric analysis confirmed that STAT1 was phosphorylated in infected IMs and Neus (Supplementary Fig. 5d) and NK cells were a major source of IFN-γ upon 17ZR101 infection (Supplementary Fig. 5e). These findings indicated both up-regulated STAT1 biosynthesis and phosphorylation were associated with hv*Kp*-induced IMs M1 polarization and Neus activation. Importantly, mice infected with 17ZR101 and intraperitoneally administrated with STAT1 inhibitor, Fludarabine (F-ara), protected 50% of mice from death within 48 h (Fig. 1k), and significantly reduce the serum IFN-γ level at 12 hpi (Fig. 1l). Together, these results confirmed that hv*Kp*-induced cytokine storm was at least partially STAT1-dependent, which is consistent with its role as the pivotal mediator of M1 macrophage polarization.^7^

Since hv*Kp* infection led to mortality by inducing cytokine storm, we then tested the therapeutic value of non-steroidal anti-inflammatory drugs (NSAIDs) on hv*Kp*-infected mice. Administration of acetylsalicylic acid (ASA), an inhibitor of cyclooxygenase-1 enzyme, which controls the production of thromboxane A2 and proinflammatory prostaglandins,^8^ protected 100% of the mice infected by 17ZR101 within 48 h (Fig. 1m), suggesting that reversing cytokine storm could protect mortality caused by hv*Kp* infection, which further confirming the key role of cytokine storm on mortality caused by hv*Kp* infection. Consistently, ASA treatment was shown to suppress IMs and Neus infiltration into the lungs, inhibit M1 macrophage polarization during hv*Kp* infection and drastically reduce serum cytokine production. Furthermore, ASA treatment was shown to inhibit *Stat1* expressions (−1.056) and down-regulate expression of three STAT1-regulated chemokines, namely *Cxcl9* (−2.118), *Cxcl10* (−3.590) and *Cxcl11*(−2.098) (Supplementary Fig. 6), confirming that STAT1 is a key transcriptional factor involved in hv*Kp*-induced IMs polarization, Neus recruitment and production of the cytokine storm.

ASA could protect against sepsis shock due to cytokine storm, but it is unable to eradicate hv*Kp* in the host. We then explore a combination therapy of ASA and antibiotics, which could not only save patients from sepsis shock caused by severe hv*Kp* infection, but also eradicate bacteria in the host by antibiotics. The treatment with a combination of ceftazidime (CAZ, 8 mg kg^−1^)—avibactam (AVI, 4 mg kg^−1^) could protect mice infected by 10^4^ CFU of 17ZR101(Supplementary Fig. 7a, b), while not for mice infected by ~10^7^ CFU with all mice died within 14 h (Fig. 1m). However, the combination of ASA (100 mg kg^−1^) with the same dose of antibiotic combination robustly rescued mice from death within 24 h, suggesting the synergistic anti-inflammatory drugs and antibiotics is both required for this process (Fig. 1n). Further analysis showed that ASA could suppress IMs infiltration (Supplementary Fig. 7c), but without contributing to bacteria clearance (Fig. 1o), indicating an anti-inflammatory role of ASA. Antibiotic (CAZ-AVI) alone could reduce the bacteria burden, but not enough to prevent mortality; even the bacterial burden was slightly higher for ASA and CAZ-AVI combinational treatment when compared to CAZ-AVI, mice were still alive suggesting the importance of the role of ASA on reversing cytokine storm (Fig. 1m, Supplementary Fig. 7d). Together, these results confirmed that the combination of ASA and antibiotics was more efficient in treating severe hv*Kp* infection.

## Supplementary information

Supplementary materials

## Data Availability

All data and materials presented in this study are available on request.
