# Supplementary material for: Molecular mechanisms underlying the high mortality of hypervirulent Klebsiella pneumoniae and its effective therapy development
Source: Signal Transduct Target Ther. 2023 May 30;8:221. doi: 10.1038/s41392-023-01490-9 (PMC10227003; doi:10.1038/s41392-023-01490-9)
Supplement: Supplementary file 1 — Supplementary materials [file 41392_2023_1490_MOESM1_ESM.docx]

Supplementary Materials for

**Molecular mechanisms underlying the high mortality of hypervirulent *Klebsiella pneumoniae* and its effective therapy development**

**Authors:** Qi Xu^a^, Miaomiao Xie^a^, Xiaoxuan Liu^a^, Heng Heng^a^, Han Wang^a^, Chen Yang^a^, Edward Wai-Chi Chan^b,c^, Rong Zhang^d^, Guan Yang^a,*^, Sheng Chen^b,c*^

**Affiliation:** ^a^ Department of Infectious Diseases and Public Health, Jockey Club College of Veterinary Medicine and Life Sciences, City University of Hong Kong, Kowloon, Hong Kong, China; ^b^Department of Food Science and Nutrition, The Hong Kong Polytechnic University, Hom Hung, Kowloon, Hong Kong, China; ^c^ State Key Lab of Chemical Biology and Drug Discovery, Department of Applied Biology and Chemical Technology, The Hong Kong Polytechnic University, Kowloon, Hong Kong, China; ^d^ Department of Clinical Laboratory, Second Affiliated Hospital of Zhejiang University, School of Medicine, Zhejiang, Hangzhou, China.

*To whom correspondence should be addressed: Dr. Guan Yang, Department of Infectious Diseases and Public Health, City University of Hong Kong, Kowloon, Hong Kong, China. Email: [gyang25@cityu.edu.hk](mailto:gyang25@cityu.edu.hk); Tel: +852 3442-4952 and Dr. Sheng Chen, Department of Food Science and Nutrition, The Hong Kong Polytechnic University, Hom Hung, Kowloon, Hong Kong, China. Email: [sheng.chen@polyu.edu.hk](mailto:sheng.chen@polyu.edu.hk); Tel: +852 3400-8619.

**This PDF file includes:**

Materials and Methods

Discussion

Supplementary figures S1-S8

**Materials and methods**

***Bacterial strains***

Two clinical *K. pneumoniae* isolates (17ZR101 and HKU1) were used in this study. Strain 17ZR101 was recovered from a 43-year-old female patient in the ICU of The Second Affiliated Hospital of Zhejiang University in 2017. The patient had undergone a tracheostomy and then subjected to tracheal intubation. She then developed symptoms of infection and *K. pneumoniae.* strain 17ZR101 was isolated from the secretion fluid of tracheal intubation. The patient was treated with meropenem and tigecycline and recovered eventually. Strain HKU1 was isolated from a blood sample of a patient in a hospital in Hong Kong. The species identity of these strains was determined by the Vitek 2 system (bioMérieux, France) and confirmed by the MALDI-TOF MS apparatus (Bruker, Germany). Phenotypic characterization and whole genome sequencing were conducted on these two strains. A string test was then performed by stretching bacterial colonies grown on sheep blood agar plates through the use of an inoculation loop. A ST11 carbapenem-resistant, hypervirulent *K. pneumoniae* strain HvKP4 and a typical ST23 K1 HvKP strain HvKP1088, which were demonstrated to be HvKP in our previous reports, were used as hypervirulence control^1,2^.

***Antibiotic susceptibility test***

The antimicrobial susceptibility of the two *Klebsiella* strains was determined by the microdilution method according to the guidelines recommended by the Clinical and Laboratory Standards Institute^3^. *Escherichia coli* strain ATCC 25922 was included as a quality control strain. All tests were performed in duplicate, and each test included three biological replicates.

***Generation of virulence plasmid-cured strain and conjugation assay***

Plasmid-cured strain 17ZR101-PC was generated from 17ZR101 by following a previously described protocol^4^. To screen for the virulence plasmid-cured strain, tellurite sensitivity assay was performed, followed by PCR test to determine if the marker gene *rmpA2* in the virulence plasmid was not present. The MIC profiles of the transconjugants were also determined for differentiation between transconjugants and the donor strains. Conjugation was also performed by using virulence plasmid harboring *E. coli* transconjugant 16ZR187-TC1 as donor^5^ and 17ZR101-PC as recipients. MacConkey agar plates containing 4 µg mL^−1^ K_2_TeO_3_ and 1 µg mL^−1^ meropenem were used to select transconjugants. The presence of *rmpA2* as a marker gene of virulence plasmid in transconjugants was determined by PCR. To further confirm that the virulence plasmid had been cured and acquisition of virulence plasmid by 17ZR101-PC, S1 and XbaI- nuclease pulsed-field gel electrophoresis (XbaI -PFGE) were performed.

***Animal experiments***

All animal experiments were approved by the Animal Ethics Committee of the City University of Hong Kong and followed the guidelines of the Institutional Laboratory Animal Research Unit. Six- to eight-week-old animals of both sexes were used in this study. For the sepsis model, mice were intravenously injected with ~10^4^ colony-forming units (CFU) of the test stain resuspended in 200 μL PBS. At 12 hpi, mice organ samples were collected for bacteria burden measurement, scRNA-Seq/RNA-Seq, western blotting, flow cytometry analysis, and serum was collected for cytokine production analysis. For the pneumonia model, mice were anesthetized by inhaling isoflurane, and ~10^6^ CFU of the strain resuspended 20 μL PBS were dripped into the mouse’s nasal cavity. The heads of the mice were held upright for 30s afterward. At 24 hpi, lung samples were collected for flow cytometry analysis.

***Preparation of cells from lung and spleen***

Cells collected from different tissues of the test animals were subjected to flow cytometry analysis as described previously^6^. Cells from spleens were obtained by mashing the organ through a 70-μm cell strainer and collected in a tube containing RPMI 1640 medium supplemented with 5% fetal bovine serum. To prepare lung cells, lung tissues were excised and incubated in HBSS containing 1 x HEPES and Collagenase type I. The tissue fragments were forced through a 70-μm strainer as described above. Red blood cells were lysed with ACK lysing buffer.

***Measurement of bacterial burden in various organs of the test animals***

Ten-fold dilutions of tissue homogenate collected were prepared and spread onto MacConkey agar plates containing 2 μg mL^-1^ cefotaxime to determine the bacterial load in different organs of the infected animals. To determine the number of intracellular bacteria, cells prepared for flow cytometry from lungs and spleens were collected and then incubated with 300 µg mL^-1^ amikacin to kill the extracellular bacteria. The number of total and intracellular bacteria was counted and presented as the number of CFU g^-1^ tissue.

***Single-cell sample preparation, data preprocessing and cell-type determination***

Lung cell suspension prepared by collagenase digestion and mashing against cell strainer was sent to BGI TECH SOLUTIONS (HONG KONG) CO., LIMITED for sequencing. Routine procedures including sample processing, library generation, cleaned data filtration, and alignment were performed by BGI. Analysis of scRNA-seq data was performed using the package Seurat (version 4.1.1). We retain valid cells based on a number of genes, mitochondrial RNA percentage, and number of UMIs (unique molecular identifiers. The raw counts were then normalized, and highly variable genes were calculated in all datasets with default parameters. We performed Seurat’s standard data integration process based on the identification of anchor cells between the two data sets. After integration, data scaling, PCA, dimensionality reduction (using UMAPs), and clustering were applied for cluster identification and data visualization. Differential expression analysis (DEA) for all clusters was performed to determine their marker genes. Marker genes of all clusters were selected based on a normalized RNA expression value. that being >0.25 log-fold higher than the mean expression value in the other sub-clusters, and with a detectable expression in > 25% of all cells from the corresponding cells. To assign identities of clusters, we listed the established lung cell types with classical markers and RNA markers and then annotated the refined clusters. Genes expressed with |log2 fold-change| >1, and with adjusted *P*-value <0.05 were considered significantly differentially expressed. Differentially expressed genes were displayed as volcano plots using EnhancedVolcano 1.4.0. Raw sequencing reads are available at the NCBI Gene Expression Omnibus (GEO) under the accession numbers GSE220594.

***RNA extraction and real-time quantitative PCR analysis***

RNA was extracted from lung samples collected by homogenization in TRIzol reagent (15596026, Thermo Fisher Scientific), followed by chloroform extraction and isopropanol precipitation. The extracted RNA was reverse-transcribed into cDNA by using a SuperScript^®^ III First-Strand Synthesis SuperMix kit (11752050, ThermoFisher Scientific). Real-time quantitative PCR was performed by using a QuantStudio™ 7 Pro Real-Time PCR System, following the manufacturer’s instructions. Primers used in qPCR are listed in **Supplementary Table 2**. cDNA samples were tested in duplicates, and the relative amount of mRNA in different samples was determined by the comparative threshold cycle (ΔΔCT) method, using the glyceraldehyde-3-phosphate dehydrogenase gene (*Gapdh*) for normalization.

***Cytokine analysis***

Mice serum was collected in all experiment unless otherwise indicated.The serum level of IL-6 of the test animals was measured by using the IL-6 Mouse Uncoated ELISA Kit (Thermo fisher scientific, **88-7064-88),** IL-1β level by IL-1 beta Mouse Uncoated ELISA Kit (Thermo fisher scientific, **88-7013-86) and** that of IFN-γ was measured by using the IFN-γ **Mouse Uncoated ELISA Kit (**Thermo fisher scientific, **88-7314-88) according to instructions of the manufacturer.**

***Flow cytometry***

Dead cells were excluded from the analysis by propidium iodide (Sigma-Aldrich Corporation, P4170-1G) or ghost dye^TM^ violet 510 (Tonbo Biosciences, 13-0870-T100) staining in all flow cytometry experiments. Fluorescently labeled mAbs against mouse CD45, CD11b, CD206, CD80, Ly6G, CD11c, SiglecF, Ly6C, and appropriate isotype controls were obtained from Biolegend. For intracellular cytokine measurement, cells were incubated with Cell Activation Cocktail (BioLegend, 423303) at 37 °C in the dark for 4 h. Anti-IFN-γ-PE antibody (eBioscience, 12-7177-81) was used to determine the intracellular expression of IFN-γ.  For p-STAT1 measurement, the cells were fixed with IC fixation buffer (eBioscience, 00-8222-49) for 30 min and then suspended in 1 mL of methanol to permeabilize the cell membrane, left to stand for 10 min on ice. At the time of p-STAT1 measurement, the cell suspensions were washed with FACS buffer and then stained with PE-anti-STAT1 Phospho antibody (Biolegend, 686404). Flow cytometric analyses were performed using a BD FACSCelesta^TM^ flow cytometer (BD Bioscience). The acquired data were analyzed by the FlowJo software (Version 10.0.7, Treestar, Palo Alto, CA).

***Western blotting***

The lung tissues of the test animals were homogenized in lysis buffer, which contained RIPA buffer, protease, and phosphatase inhibitor (Roche). Protein concentration was measured by performing the Bradford Assay (Bio-Rad Laboratories). Briefly, 10 μL of the cell lysates were resolved by standard 12% SDS-PAGE gel and electroblotted onto 0.2 mm nitrocellulose membrane (Bio-Rad) using a semi-dry transfer unit (Bio-Rad). The membranes were blocked with 5% milk in TBST for 1 h at room temperature, and then incubated overnight at 4°C with the following primary antibodies diluted in 5% BSA in TBST. Primary antibodies against STAT1 (1:4000, #9172T), phospho-STAT1 (1:4000, #7649T) and MAPK p38 (1:4000, #8690T) were obtained from Cell Signaling Technologies. β-actin (1:4000, #ab119716) was obtained from Abcam. Immunoreactive bands were visualized by incubation with goat anti-rabbit immunoglobulins (1:5000, Abcam, #ab205718). Each experiment was repeated at least twice.

***RNA-Seq analysis***

Total RNA was extracted from lung samples collected at 12 hpi by homogenization in TRIzol reagent, followed by chloroform extraction and isopropanol precipitation. DNA was removed by DNase treatment (TURBO DNA-free^TM^ Kit). RNA samples were sent to Novogene (HONG KONG) Company Limited for sequencing. Routine procedures of mRNA purification and library generation were performed by Novogene. Sequencing reads alignment was performed by using HISAT2. Count-aligned reads and quantification were calculated based on [exon](https://www.sciencedirect.com/topics/agricultural-and-biological-sciences/exons) regions using FeatureCounts. Significantly changed genes (FPKM ≥ 1 in either Sham-, 17ZR101-, HKU1-, or 17ZR101+ASA-treatment group, |Log2Fold Change| > 1, padj < 0.01) were identified by DESeq2 analysis. Gene Set Enrichment Analysis was performed for [gene](https://www.sciencedirect.com/topics/immunology-and-microbiology/gene-ontology) ontology enrichment analysis. The raw RNA-seq data has been deposited in NCBI database under the accession number PRJNA851242.

***Drug treatment***

In ASA, naproxen (NPXS), dexamethasone (DXMS), and F-ara treatment experiments, mice challenged by 17ZR101 were given intraperitoneally 100 mg kg^-1^ ASA, 50 mg kg^-1^ NPXS, 10 mg kg^-1^ DXMS and 40 mg kg^-1^ F-ara at 3 hpi, another group treated with an equal volume of PBS or 200 μL of corn oil solution was included as a control group*.* In ASA and CAZ/AVI combination treatment experiment, mice were given intraperitoneally 100 mg kg^-1^ ASA plus 8 mg kg^-1^ CAZ and 4 mg kg^-1^ AVI or 8 mg kg^-1^ CAZ and 4 mg kg^-1^ AVI at 1 hpi. The health status and weight of all mice were observed and recorded.

***Statistical Analysis***

Statistical analysis of data obtained in this work was performed by means of using Graphpad Prism 6.0 (GraphPad Software, La Jolla California USA, www.graphpad. com). Statistical analyses on normally distributed data sets were performed using one-way ANOVA with Tukey’s correction for multiple comparisons. The log-rank test was used for comparing survival rate in animal experiments. P values < 0.05 was considered significant. Unless otherwise indicated, the survival curve of mice in animal experiments and results of flow cytometry analysis were representative of at least two independent experiments.

**Discussion**

In this paper, we investigated why hv*Kp* infection is often associated with high mortality. Unlike c*Kp* which can be readily cleared by host defense system during infection, hv*Kp* exhibits resistance to host killing and is therefore able to continuously stimulate the host immune response during the infection process. Our data show that the persistence of the hv*Kp* strain in the host triggers the onset of a cytokine storm through activation of the STAT1 pathway. The cytokine storm eventually leads to a septic shock, which has a high risk of death. In this work, we showed that this hv*Kp*-triggered cytokine storm could be suppressed by immune-suppressants such as ASA. The M1 polarization is involved in protection against acute infections; yet uncontrolled activation of M1 macrophages activation may be responsible for causing the disease symptoms during *Kp* infection^7^. Our results showed that hv*Kp* induced excessive M1 polarization and neutrophil infiltration. However, this infiltration did not result in hv*Kp* clearance, indicating that hv*Kp* could co-exist with phagocytes. We therefore conclude that the ability to resist killing by phagocytes is the underlying mechanism by which hv*Kp* exhibits high-level virulence and a high potential of causing the death of the host during infection. This unique feature of hv*Kp* is attributed to the abundance of the capsule and the large number of siderophore systems possessed by such strains^8^. It was reported that hv*Kp* strains produce a very thick capsule that effectively protects the bacterium from the hostile environment^9^ and various siderophores help acquire iron from iron-depleted environments^10^. In particular, aerobactin contributes to better growth and survival of hv*Kp* *in vivo*^11^. In hv*Kp* strain 17ZR101, the capsule regulator *rmpC* and the aerobactin-coding genes *iucABCD* are located in the virulence plasmid. Our results showed that the virulence plasmid is essential for the pathogenesis of hv*Kp* by conferring better survival in host. Several other studies also demonstrated that neutrophil extracellular traps escaping^12^ and neutrophil-mediated phagocytosis resistance^13^ also contributed to the ability of hv*Kp* to resist killing by phagocytes. In this study, we found that hv*Kp* was partially internalized but not eradicated, confirming that hv*Kp* developed a defensive mechanism that can modulate phagocytosis and suppress killing by the immune cells of the host. Consistently, a previous study showed that *Kp* actively manipulated phagosome maturation and phagolysosome fusion to its advantage^14^. Future studies should focus on virulence components essential for hv*Kp* pathogenesis and how these factors manipulate the host immune system.

Cytokine storm is a fast-developing, life-threatening clinical condition in which over-production of inflammatory cytokines and excessive activation of immune cells cause a range of damages in the host, with a high risk of death if left untreated^15^. On the other hand, cytokine storm is known to be associated with infections caused by influenza and various other viruses, bacteria, or fungi, with sepsis being a condition commonly associated with the onset of cytokine storm^16^. Multiple reports suggested the formation of cytokine storms in patients infected by SARS-CoV and MERS-CoV^16^. One possible mechanism linking cytokine storm to organ damage is the process of cell death. Among the programmed cell death pathways, pyroptosis, apoptosis, and necroptosis have been best characterized. Depending upon the stimulus encountered, cells can experience extensive crosstalk, leading to PANoptosis. Studies have reported that SARS-CoV-2 induced PANoptosis in human and murine macrophages^17,18^. Similarly, *Kp* infection was also found to cause pyroptosis^19^, apoptosis^20^ and necroptosis^21^. However, whether PANoptosis is directly involved in the onset of septic shock resulting in cytokine storm during hv*Kp* infection, and the detailed regulatory mechanisms concerned, entails further investigation.

**Supplementary Figures**


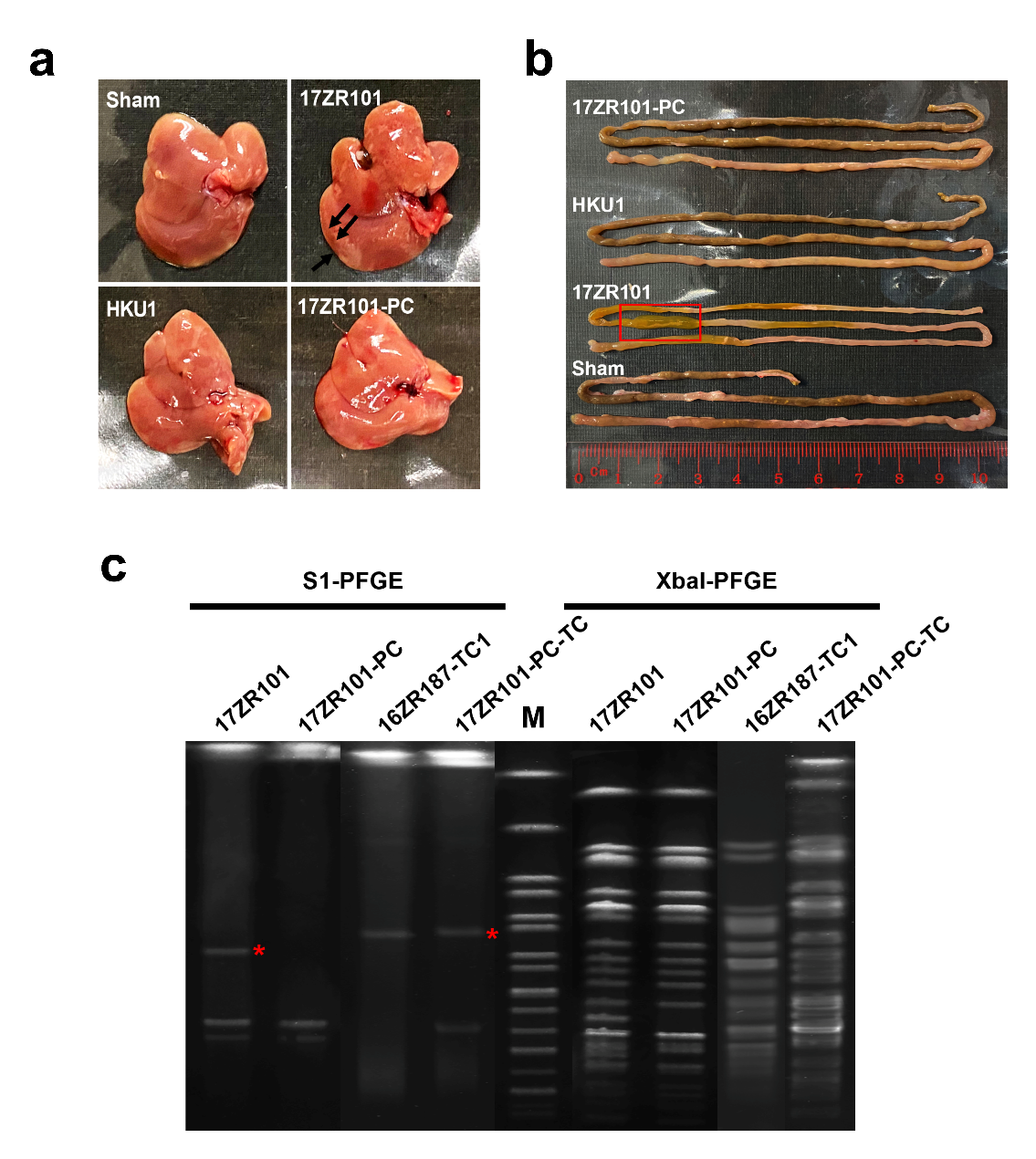


**Supplementary Fig. 1. Virulence plasmid curing altered the morphology of liver and small intestine in mice of hv*Kp* infection.**

**(a)** Changes in liver morphology among mice infected by different *Klebsiella* strains. **(b)** Hv*Kp* infection caused thinning of the intestinal walls and production of a large amount of yellow mucus-like substance in the intestine. **(c)** Validation of the virulence plasmid curing and conjugation in 17ZR101 by S1- and XbaI-PFGE. “*” indicates the virulence plasmid to be cured and conjugated.


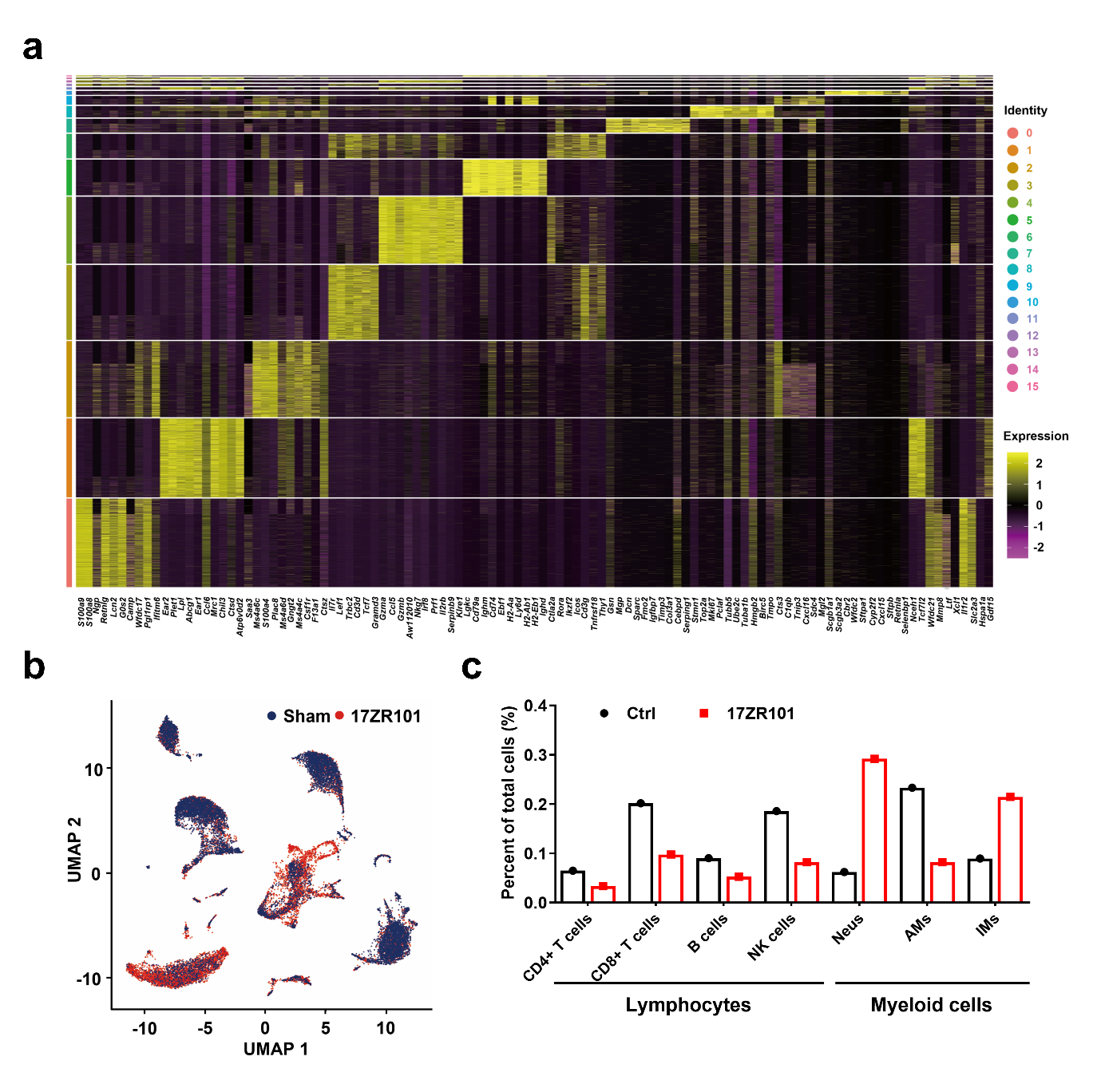


**Supplementary Fig. 2. Differential expression analysis comparing cells from Sham- and 17ZR101-infected lungs.**

**(a)** Heatmap was shown representing up- and down-regulated genes in different groups of cells. **(b)** Origins of cells with same embedding as in **Fig. 1e**. **(c)** Percentage comparison of seven main type of cells (CD4^+^ T cell, CD8^+^ T cell, NK cell, B cell, Neutrophil, Alveolar macrophage, and interstitial macrophage identified by scRNA-Seq.


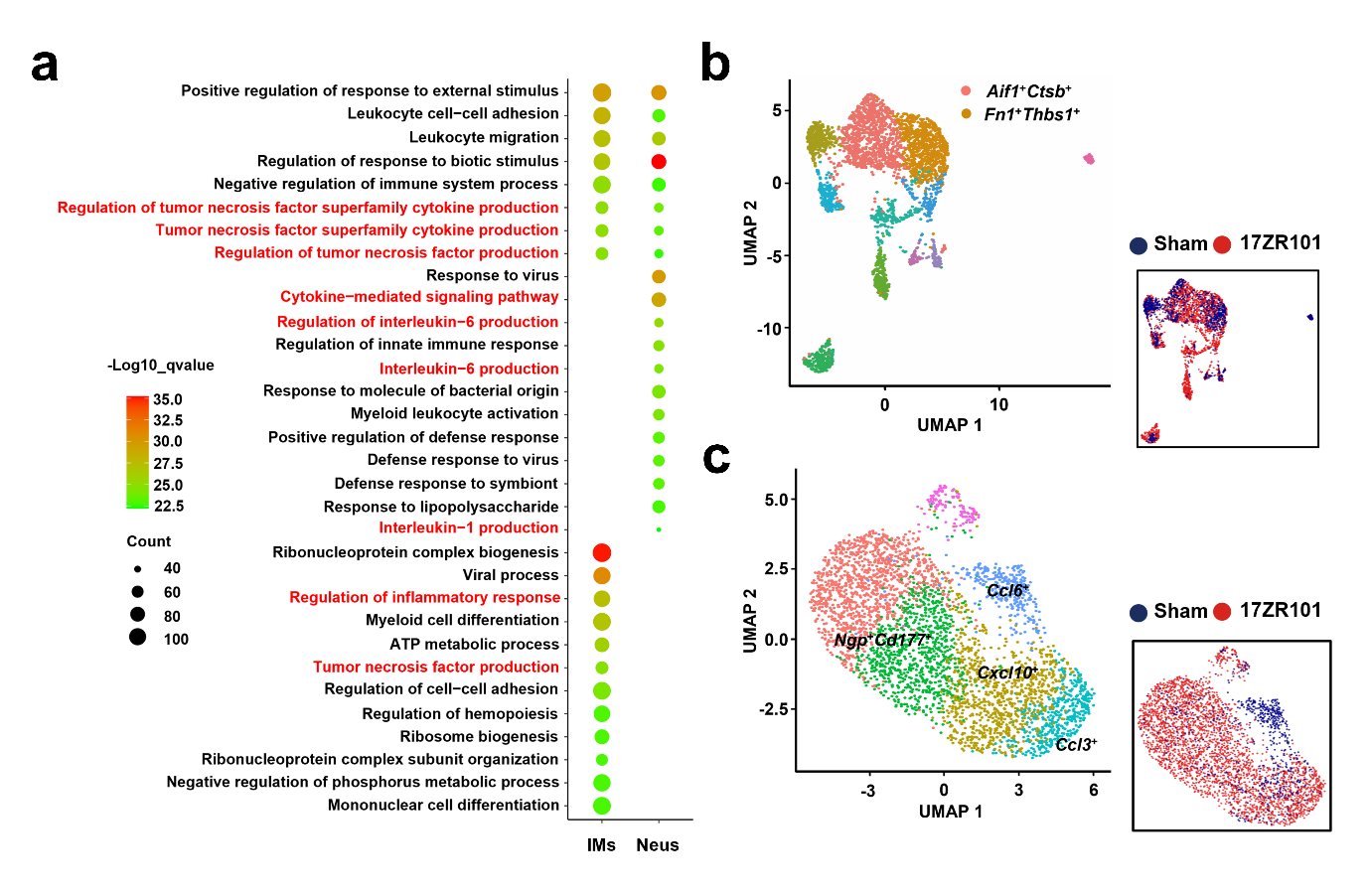


**Supplementary Fig. 3. ScRNA-Seq revealed the pro-inflammatory state of IMs and Neus.**

**(a)** Go enrichment analysis of changing pathways in IMs and Neus. **(b)** UMAP of single cell profile coded for IMs subsets. **(c)** UMAP of single cell profile coded for Neus subsets.


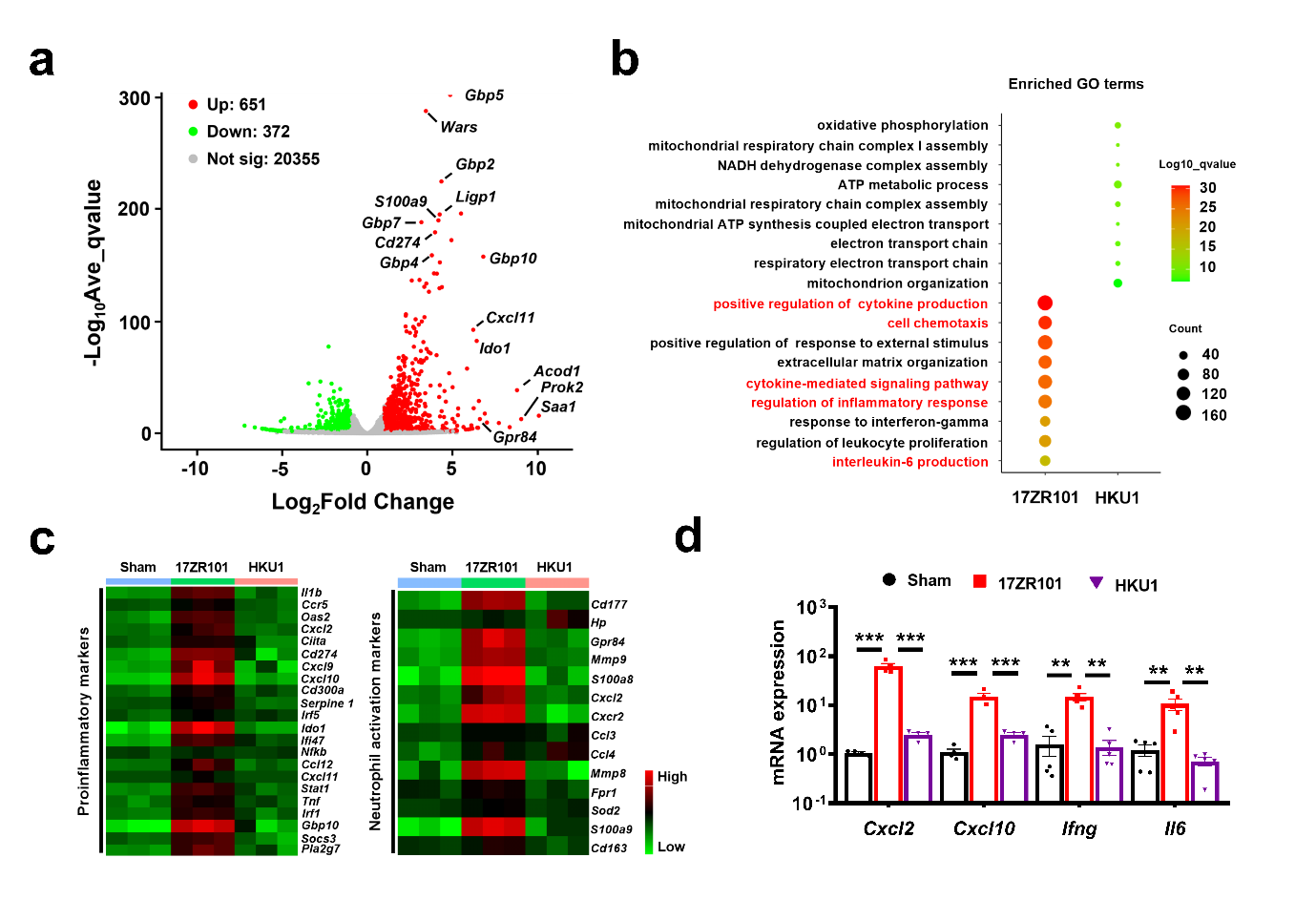


**Supplementary Fig. 4. Hv*Kp* induces cytokine storm in the host.**

**(a)** Volcano plot of RNA-Seq gene counts in total lung cells obtained from Sham- and 17ZR101-infected mice (n = 3 mice per group). **(b)** Enriched GO analysis showing changed pathways in 17ZR101- and HKU1-infected lung cells. **(c)** Heatmap depicting the differential expression patterns of the proinflammatory and neutrophil activation marker gene clusters in the Sham-, 17ZR101- and HKU1-infected groups of mice. **(d)** Fold-change in genes of the M1 markers, proinflammatory cytokines in lung cells which exhibited altered gene expression patterns in qPCR analysis of 17ZR101-treated mice, with Sham- and HKU1-treated lung cells being the control (n = 5 per group). ***p* < 0.01, ****p* < 0.001.


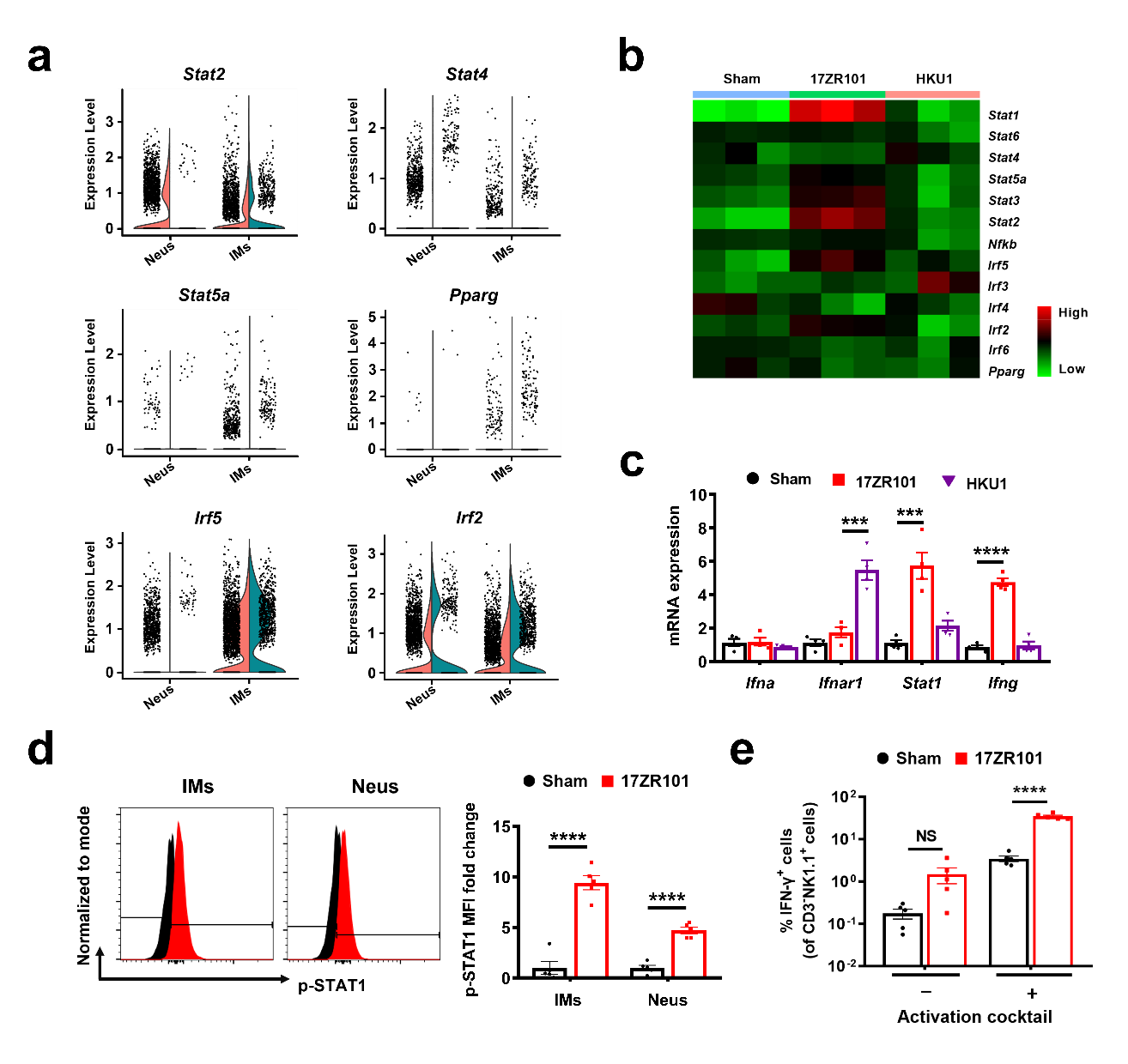


**Supplementary Fig. 5. Transcriptional regulation of M1 polarization.**

**(a)** Expression of *Stat2*, *Stat5a*, *Stat4*, *Pparg*, *Irf5* and *Irf2* in Neus and IMs were identified by scRNA-Seq. **(****b)** Heatmap of transcription factors involved in macrophage polarization. **(c)** Fold-change in gene counts of *Ifna*, *Ifnar1*, *Stat1,* and *Ifng* in 17ZR101- compared with Sham- and HKU1-infected lung cells as determined by RNA-seq. n=5. **(d)** Representative of histogram and MFI change of p-STAT1 on IMs and Neus were shown. **(e)** Total lung cells from Sham- and 17ZR101-infected mice (12 hpi) were cultured with activation cocktail. After 4 h of culture, cells were harvested and intracellular staining was performed (IFN-γ), and cytokine-producing NK1.1^+^ cells were analyzed by flow cytometry.


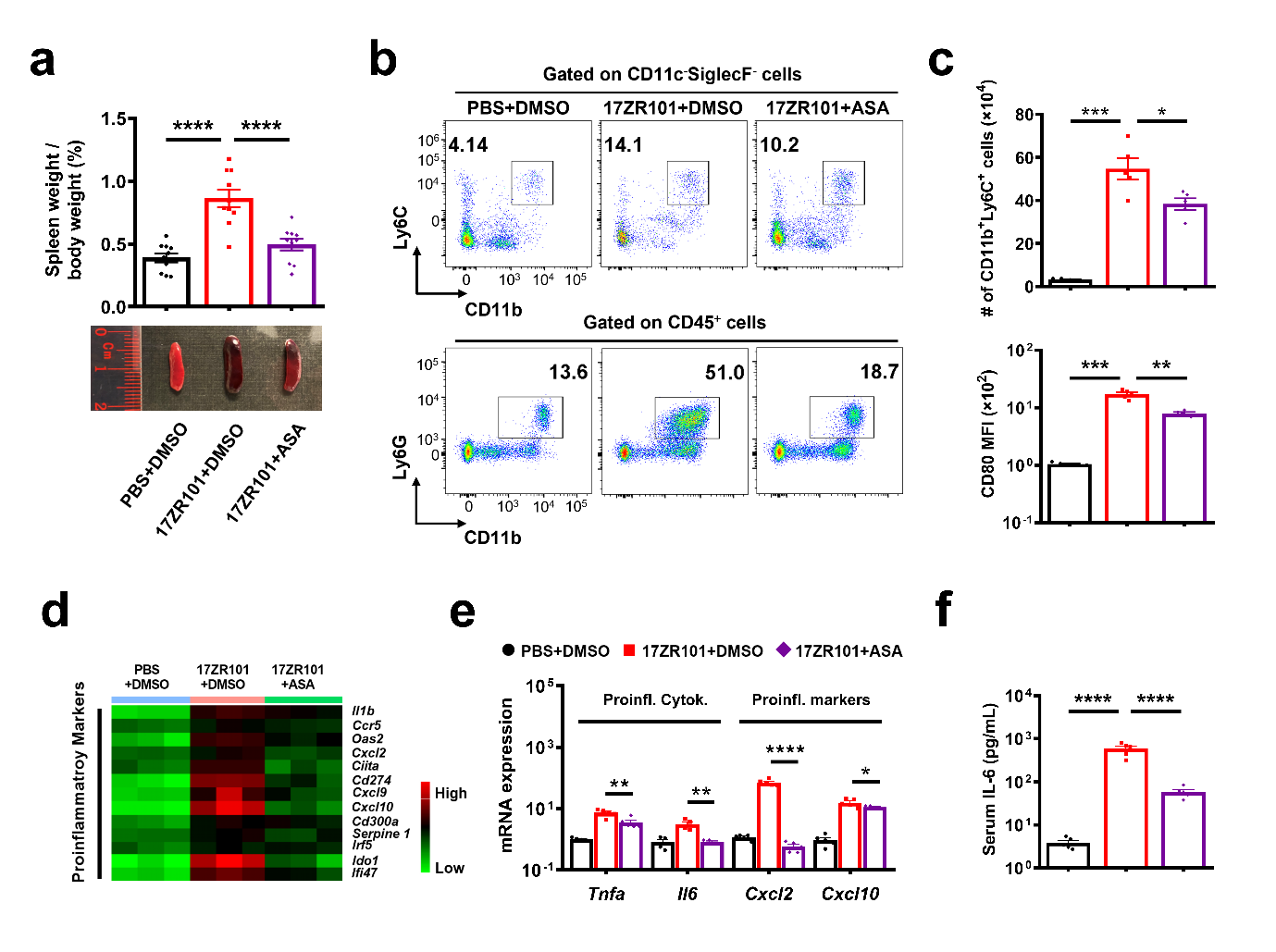


**Supplementary Fig. 6. ASA suppresses the cytokine storm induced by hv*Kp*.**

**(a)** Weight of spleen normalized to body weight and representative images of spleens harvested from Sham, 17ZR101, and 17ZR101+ASA treated mice, n=10. **(b)** Flow cytometry analysis of IMs and Neus in total lung cells of 17ZR01-infected mice with or without ASA treatment was shown. **(c)** Quantification of IMs in lungs and CD80 MFI on the surface of IMs were shown. **(d)** Comparative analysis of individual gene transcript expression in indicated groups for proinflammatory markers. n=3. **(e)** Fold-change in expression level of genes related to macrophage polarization and inflammation in lung cells of 17ZR101+ASA-treated mice, with lung cells of Sham- and 17ZR101-treated mice as control. n=5. (**f**) Quantification of serum IL-6 levels measured by ELISA kit. **p* < 0.05, ***p* < 0.01, ****p* < 0.001, *****p* < 0.0001.


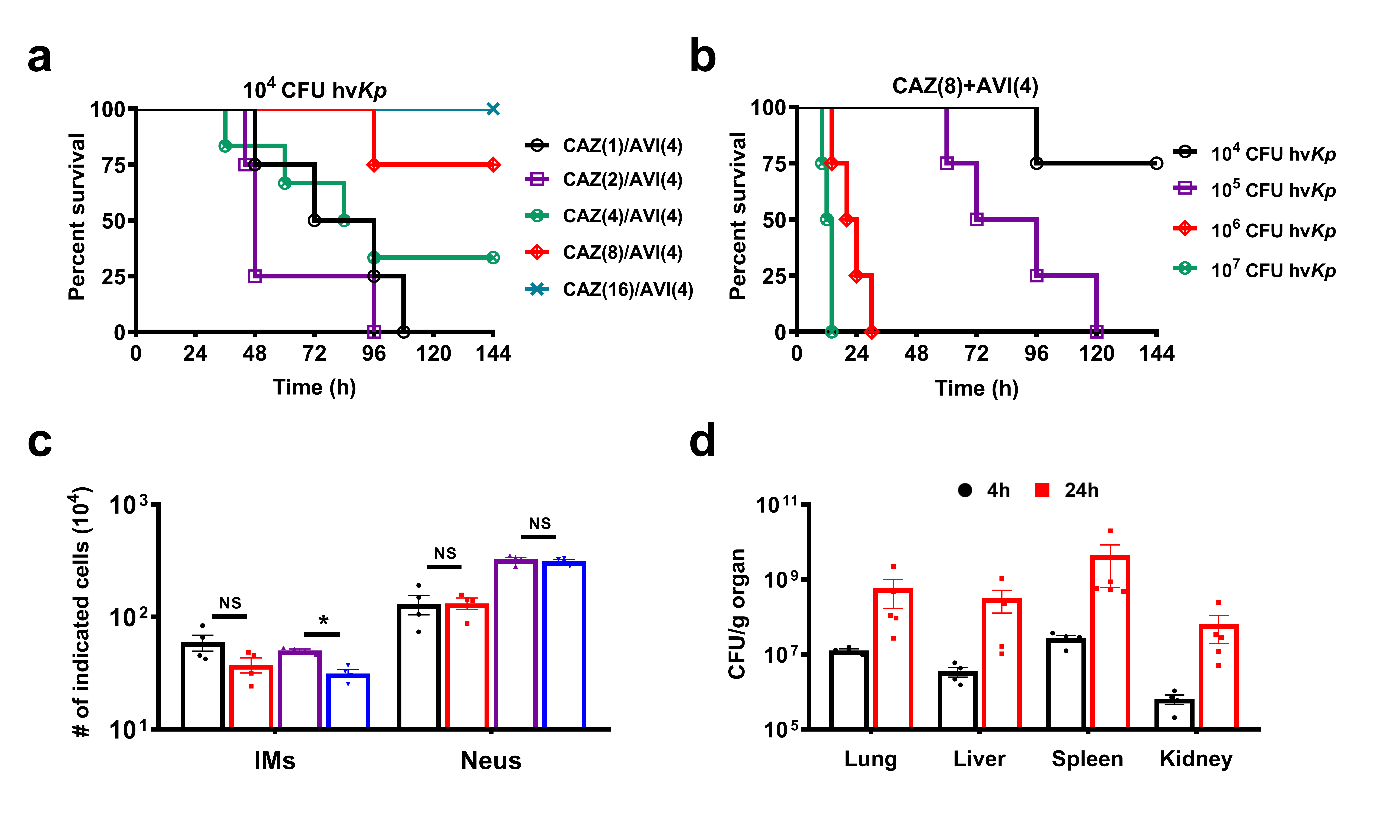


**Supplementary Fig. 7. The protection of CAZ/AVI and ASA on mice under hv*Kp* challenge.**

**(a)** C57BL6 mice were inoculated with 10^4^ CFU hv*Kp* strain and treated with indicated dose of CAZ and fixed dose of AVI at 3 hpi. The survival curve was recorded. **(b)** The Kaplan-Meier survival curve of mice infected by indicated dose of hv*Kp* strain 17ZR101 and treated with CAZ (8)-AVI (4). n = 5. **(c)** Quantification of IMs and Neus in infected lungs from **Fig. 1n** analyzed by flow cytometry at 4 hpi was shown. **(d)** C57BL6 mice were inoculated with 10^7^ CFU of indicated hv*Kp* strain 17ZR101 and treated with CAZ/AVI + ASA at 1 hpi. Bacteria burdens of infected mice at 4 and 24 hpi were recorded.


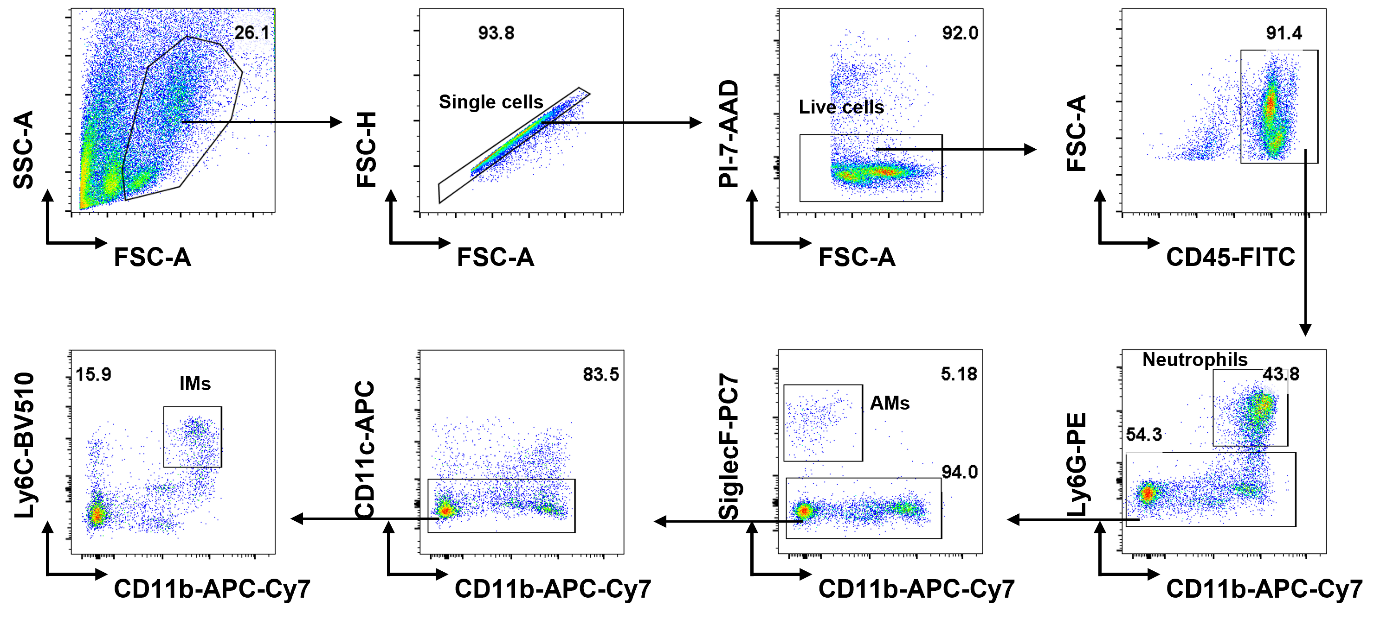


**Supplementary Fig. 8. Gating strategy to select neutrophils and interstitial macrophages for flow cytometry.**

| **Strain ID** | **MLST Type** | **Serotypes** | **Virulence genes** | **Resistance determinants** | **MIC values (µg mL^-1^)** | | | | | | | |
| --- | --- | --- | --- | --- | --- | --- | --- | --- | --- | --- | --- | --- |
|  |  |  |  |  | **IMP** | **AMK** | **CIP** | **CTX** | **CAZ** | **MRP** | **PB** | **TIG** |
| 17ZR101 | ST86 | K2 | *irp1*; *irp2*; *iucABCDiutA*; *iroBCDN*; *kugA*; *kvgS*; *mrkABCDFHIJ*; *rmpA* | *amp(H)*; *bla*KPC-2; *bla*SHV-106_;_ *oqxAB*; *qnrs1* | 32 | 1 | 1 | > 128 | > 128 | > 128 | 4 | 1 |
| HKU1 | ST716 | K9 | *mrkABCDFHIJ* | *aac (3)-IId*; *bla*CTX-M-3; *bla*SHV-26; *bla*TEM-1B; *dfrA28*; *floR2*; *fosA3*; *mph(A)*; *oqxAB*; *qnrB20*; *strA4*; *strB1*; *sul1*; *sul2*; *tet(A)* | 0.5 | 4 | 8 | > 128 | 64 | 32 | 4 | 2 |

**Supplementary Table 1. Phenotypic and genotypic characteristics of *K. pneumoniae* strains tested in this study.**

IPM, imipenem; AMK, amikacin; CIP, ciprofloxacin; CTX, cefotaxime; CAZ, ceftazidime; MRP, meropenem; PB, polymyxin B; TIG, tigecycline

| **Gene** | **Strand** | **Sequence 5’-3’** |
| --- | --- | --- |
| *Gapdh* | Forward | AGGTCGGTGTGAACGGATTTG |
|  | Reverse | TGTAGACCATGTAGTTGAGGTCA |
| *Tnfa* | Forward | CTTGGAAATAGCTCCCAGAA |
|  | Reverse | CATTTGGGAACTTCTCATCC |
| *Ifng* | Forward | GCTTTGCAGCTCTTCCTCAT |
|  | Reverse | GCAGGATTTTCATGTCACCA |
| *Cxcl2* | Forward | CAGAAGTCATAGCCACTCTCAA |
|  | Reverse | CTCCTTTCCAGGTCAGTTAGC |
| *Cxcl10* | Forward | GCTGCAACTGCATCCATATC |
|  | Reverse | GTGGCAATGATCTCAACACG |
| *Ifna* | Forward | CCTGCTGGCTGTGAGGA |
|  | Reverse | GGAAGACAGG GCTCTCCAG |
| *Ifnar1* | Forward | ACATCACCTGCCTTCACCAG |
|  | Reverse | CATGGAGCCACTGAGCTTGA |
| *Stat1* | Forward | TACGGAAAAGCAAGCGTAATCT |
|  | Reverse | TGCACATGACTTGATCCTTCAC |
| *Il-6* | Forward | ACAACCACGGCCTTCCCTAC |
|  | Reverse | TCTCATTTCCACGATTTCCCAG |

**Supplementary Table 2. Primers used for qPCR in this study.**

**Reference**

1 Gu, D. *et al.* A fatal outbreak of ST11 carbapenem-resistant hypervirulent Klebsiella pneumoniae in a Chinese hospital: a molecular epidemiological study. *The Lancet infectious diseases* **18**, 37-46 (2018).

2 Zhang, R. *et al.* Emergence of carbapenem-resistant serotype K1 hypervirulent Klebsiella pneumoniae strains in China. *Antimicrobial agents and chemotherapy* **60**, 709-711 (2016).

3 Wayne, P. A. Clinical and Laboratory Standards Institute : Performance standards for antimicrobial susceptibility testing : 20th informational supplement. *CLSI document M100-S20* (2010).

4 El-Mansi, M., Anderson, K. J., Inche, C. A., Knowles, L. K. & Platt, D. J. Isolation and curing of the Klebsiella pneumoniae large indigenous plasmid using sodium dodecyl sulphate. *Research in microbiology* **151**, 201-208 (2000).

5 Xie, M. *et al.* Conjugation of virulence plasmid in clinical Klebsiella pneumoniae strains through formation of a fusion plasmid. *Advanced biosystems* **4**, 1900239 (2020).

6 Yang, G. *et al.* Pik3c3 deficiency in myeloid cells imparts partial resistance to experimental autoimmune encephalomyelitis associated with reduced IL-1β production. *Cellular & Molecular Immunology* **18**, 2024-2039 (2021).

7 Li, Q. R. *et al.* Mechanism of chlorogenic acid in alveolar macrophage polarization in Klebsiella pneumoniae‐induced pneumonia. *Journal of Leukocyte Biology* (2021).

8 Shon, A. S., Bajwa, R. P. & Russo, T. A. Hypervirulent (hypermucoviscous) Klebsiella pneumoniae: a new and dangerous breed. *Virulence* **4**, 107-118, doi:10.4161/viru.22718 (2013).

9 Doorduijn, D. J., Rooijakkers, S. H., van Schaik, W. & Bardoel, B. W. Complement resistance mechanisms of Klebsiella pneumoniae. *Immunobiology* **221**, 1102-1109 (2016).

10 Hider, R. C. & Kong, X. Chemistry and biology of siderophores. *Natural product reports* **27**, 637-657 (2010).

11 Russo, T. A., Olson, R., MacDonald, U., Beanan, J. & Davidson, B. A. Aerobactin, but not yersiniabactin, salmochelin, or enterobactin, enables the growth/survival of hypervirulent (hypermucoviscous) Klebsiella pneumoniae ex vivo and in vivo. *Infection and immunity* **83**, 3325-3333 (2015).

12 Wang, L., Shen, D., Wu, H. & Ma, Y. Resistance of hypervirulent Klebsiella pneumoniae to both intracellular and extracellular killing of neutrophils. *PLoS One* **12**, e0173638 (2017).

13 Xu, Q., Yang, X., Chan, E. W. C. & Chen, S. The hypermucoviscosity of hypervirulent K. pneumoniae confers the ability to evade neutrophil-mediated phagocytosis. *Virulence* **12**, 2050-2059, doi:10.1080/21505594.2021.1960101 (2021).

14 Cano, V. *et al.* Klebsiella pneumoniae survives within macrophages by avoiding delivery to lysosomes. *Cell Microbiol* **17**, 1537-1560, doi:10.1111/cmi.12466 (2015).

15 Mangalmurti, N. & Hunter, C. A. Cytokine storms: understanding COVID-19. *Immunity* **53**, 19-25 (2020).

16 Chousterman, B. G., Swirski, F. K. & Weber, G. F. in *Seminars in immunopathology.* 517-528 (Springer).

17 Karki, R. *et al.* Synergism of TNF-α and IFN-γ triggers inflammatory cell death, tissue damage, and mortality in SARS-CoV-2 infection and cytokine shock syndromes. *Cell* **184**, 149-168. e117 (2021).

18 Karki, R. *et al.* ZBP1-dependent inflammatory cell death, PANoptosis, and cytokine storm disrupt IFN therapeutic efficacy during coronavirus infection. *Science Immunology*, eabo6294 (2022).

19 Ye, C. *et al.* Inappropriate use of antibiotics exacerbates inflammation through OMV-induced pyroptosis in MDR Klebsiella pneumoniae infection. *Cell reports* **36**, 109750 (2021).

20 Wang, Z. *et al.* Hypermucoviscous Klebsiella pneumoniae infections induce platelet aggregation and apoptosis and inhibit maturation of megakaryocytes. *Thrombosis Research* **171**, 45-54 (2018).

21 Yin, H. *et al.* Taurine inhibits necroptosis helps to alleviate inflammatory and injury induced by Klebsiella infection. *Veterinary Immunology and Immunopathology*, 110444 (2022).
